# The Relationship between Creativity and Mental Disorder in an African Setting***

**DOI:** 10.4103/0973-1229.77439

**Published:** 2011

**Authors:** O. Olugbile, M.P. Zachariah

**Affiliations:** * *Department of Psychiatry, Lagos State University Teaching Hospital, 1-3 Oba Akinjobi Street, GRA, Ikeja, Lagos State, Nigeria*; ** *Department of Psychiatry, Lagos State University Teaching Hospital, 1-3 Oba Akinjobi Street, GRA, Ikeja, Lagos State, Nigeria*; *** *Revised and peer reviewed version of a paper presented at an International Seminar on Mind, Brain, and Consciousness, Thane College Campus, Thane, India, January 13-15, 2010.*

**Keywords:** *Creativity*, *Cultural beliefs*, *Mental illness*, *Personality*, *Recovery*

## Abstract

**Background:**

There has for some time now been recognition that there was a relationship between exceptional creative talent and mental disorder. The works of Andreasen (2008) and others in this area have been very significant. However, most of the research has been carried out in USA and Europe. Very little has come out of Africa on the subject.

**Aim:**

To survey the beliefs of different groups within an African society, concerning the possibility of a relationship between creative talent and mental disorder. To assess creativity within a community of people with a formal diagnosis of mental disorder.

**Materials and Methods:**

Some of the mythology of the Yoruba was examined for content, concerning the behaviour of certain notable individuals and the existence of psychopathology based on modern-day criteria. The beliefs of members of the general public and mental health professionals concerning the existence of a relationship between creative talent and psychopathology were surveyed using a questionnaire designed for the project. A sample of patients with formal diagnoses of affective disorder or schizophrenia drawn from two units, the Lagos State University Teaching Hospital and the Federal Neuropsychiatric Hospital Yaba, were assessed for ‘Creativity.’

**Results:**

Although there are notable ‘eccentric’ figures in local mythology, the overwhelming majority of the people surveyed do not believe there is any relationship between creativity and mental illness. They however believe that engaging in creative activities helps the mentally ill to recover from illness. The mental health professionals, who were clinical psychologists and psychiatrists, had a significant minority who believed that a relationship does exist, and they also strongly assert that creative activity has a therapeutic effect for the mentally ill. A survey of in-patients diagnosed with schizophrenia and affective disorder failed to show a significant difference in the creativity of the two populations, as measured by the originality score of the Rorschach scale. The survey of patients is inconclusive, based on small sample size (ten patients with a diagnosis of schizophrenia, ten with bipolar affective disorder.). The linkage between formal mental disorder is only recognised by a significant minority of mental health professionals. A significant proportion of the population believe that creative activity aids recovery from mental illness. More research is required into this important subject in Africa.

## Introduction

The relationship between exceptional talent, especially ’creative’ talent, and the possibility of odd, unusual or frankly abnormal behaviour is one that has intrigued various writers over time. Creativity is a difficult subject to define. How does one quantify the talent of the novelist, or the atomic scientist who breaks new grounds in his research?

The Longman Active Study dictionary (Adrian-Vallance *et al*., 2004) defines creativity as ’… involving the use of imagination to produce new ideas or things.’

In the past, creativity tended to be equated with intelligence. It is now known that the two factors are independent, though most creative people appear to have an IQ of 120 and above (Andreasen and Glick, 1988). For the most part, the definition of creativity has been based on the perceived originality of the ’creative’ product.

More recently, attempts have been made to quantify ’creativity’ independent of the subject’s fame or intelligence quotient. This often involves a combination of an operational definition (what the person has produced) and the use of psychological markers that are said to be characteristic of creative persons (nonconforming, adventurous, sensitive, introspective, independent).

Much of the early interest of researchers focussed on a suggested relationship between creativity and schizophrenia. Lumbroso (1891) introduced the concept of ’hereditary taint’ to describe the relationship between the manifestation of exceptional talent (’genius’) in certain people and the presence of ’madness.’ In a study published in 1926, (Ellis, 1926) a researcher selected 1,020 eminent people in the U.K. They included politicians, scientists and artists. They were actually chosen more for their fame than any creative talent. The researcher found that 4.2% of his sample were ’insane’ (i.e., psychotic), 8% ’melancholic’ (depressed), 16% imprisoned and 5% had ’personality disorder.’ Brain (1948) observed that geniuses were more ’nervous’ than other people. When they became ’insane,’ the diagnosis was often ’cyclothymia.’ Juda (1949) explored the records of 113 artists and 18 scientists. He found ’personality disorder’ to be the commonest diagnosis. Artists showed alcoholism and schizophrenia, while scientists more frequently had affective disorder. Other researchers have sought to establish linkages between creativity and mental health syndromes (Murray *et al*., 2010; Moses, 2010; MacCabe *et al*., 2010; Akinola and Mendes, 2010).

A Danish study (McNeil, 1971) measured psychopathology in some creative people (who had been adopted away from an early age), and also in their biological and adoptive relatives. It found that 3% of the ’creative’ sample had psychiatric diagnosis. 28% of their biological parents also had a diagnosis, while only 5% of the adoptive parents were ill. The commonest diagnosis was ’Reactive Psychosis’ (A Scandinavian term equivalent to Affective disorder). Rust and collaborators (Rust *et al*., 1988) reported a study designed to test the traditionally assumed relationship between creativity and schizophrenia. They found a relationship between creative originality and the positive cognitive aspects of schizotypal thinking. Richard (1988), working from Harvard University, set out to answer the intriguing question-is there a compensatory advantage in Manic-Depressive Illness? In entertaining such a possibility, he had in mind the examples of sickle cell disease, where the heterozygote is supposed to enjoy relative immunity from malaria. He selected a sample of Manic-depressives, cyclothymes and normal first-degree relatives along with matched controls. He measured their creativity using a ’Lifetime Creativity Scale.’ He found creativity to be higher among the test sample than the controls. There was also more creativity among normal first-degree relatives than among the ill patients themselves, with those diagnosed as cyclothymic being in-between. The conclusion was that the liability to Manic-Depressive illness carries an advantage for Creativity, especially among individuals who are not actively ill.

Working from another direction, another researcher (Jamison, 1989) took a sample of 47 famous living British Writers and Artists. They were people who had won major awards such as the Booker Prize, or were distinguished members of the Royal Academy of Arts. She found that 38% of them had received treatment for affective disorder (antidepressant, lithium and/or hospitalisation). Poets and novelists were particularly prone to mood swings, whereas visual artists were less vulnerable. It was generally recorded that the writers had intense creative episodes lasting 1 to 4 weeks, marked by increased enthusiasm, increased energy and self-confidence and high speed of mental association. These resemble the mood and cognitive components of Hypomania, without the behavioural nuisance attributes of talkativeness, hypersexuality and excessive spending. In a similar vein, Andreasen in Iowa (Andreasen, 1987), and over the years, collected a sample of famous writers who came to work on the University faculty. She found that 80% of the writers had had an episode of affective illness at sometime, compared with 30% of a control sample. 43% of the writers had bipolar disease. There was also a higher incidence of illness and creativity in the writers’ first-degree relatives (see also, Andreasen, 2011).

Africa is a continent on which the issue of creative expression plays a central cultural role in the everyday life of the people. Although there are myths that imply an expectation and tolerance of odd behaviour among powerful creative figures, no formal study of the relationship between creativity has been carried out on the continent.

The present research is an attempt to add an African dimension to the discussion.

## Materials and Methods

The study was carried out in two parts. The first part involved a survey of a sample of the population in South-West Nigeria on their perception of the possible connection between creativity and mental illness. The following three groups of participants were surveyed: (i) 50 members of the professional mental health community comprising of psychiatrists, psychologists and mental health nurses; (ii) An urban sample comprising of 200 residents of Lagos city and (iii) 100 participants from a rural area in South West Nigeria.

The second part of the study involved assessing the creativity, specifically originality, of 10 participants diagnosed as schizophrenics and 10 manic depressive participants. The identification of these participants was based on the administration of the Mini Mental State Examination protocol, which was carried out by resident doctors in two psychiatric facilities in Lagos.

### Instruments

1. The perception of the relationship between creativity and mental illness was surveyed using two versions of a questionnaire specifically customised for the mental health professionals on one hand, and the general public on the other. The questionnaire for the rural sample was translated into Yoruba for easy understandability. The following perceptual variable were surveyed using the questionnaire:

- Perception of relationship between creativity and mental illness
- The nature of the relationship, if any
- Creative persons’ proneness to mental illness
- Whether mentally ill persons are more creative than others
- The potential for the creative process to have therapeutic effect for mentally ill persons

2. The Rorschach Inkblot test was administered to participants diagnosed as schizophrenic and manic depressive psychotics. Specifically, the Rorschach ’O’ originality score was computed as an index of creative perception. The participants were placed in diagnostic categories based on case note diagnosis and the Mini Mental State Examination.

### Procedure

Two researchers surveyed the professional mental health group of participants. They included graduate students, psychiatrists, psychiatry resident doctors and psychiatric nurses. Urban and rural members of the general public were surveyed by two collaborators (a social worker and a psychiatric nurse). The Rorschach Ink Blot test was administered and scored by the clinical psychologist member of the research team.

### Analysis of data

The nonparametric techniques of Chi square and the Man Whitney test were used to analyse the data. The SPSS statistical package was used for this purpose.

## Results

### General comments

1. There appears to be notable difference in the way mental health professionals perceive creative persons’ proneness to mental illness compared with both the urban and rural general public participant groups. These groups distinctly perceive creative persons as not being prone to mental illnesses, whereas the mental health professional group seems to be divided in their perspective. A sizable minority of them appear to feel that creative persons are prone to develop mental illnesses [Tables 1–3].
2. Rural participants do not see any notable relationship between creativity and mental illnesses, whereas both professional and urban groups appear to perceive a significant relationship.
3. There appears to be a strong consensus of opinion that mentally ill persons are not significantly more creative than others.
4. All groups uniformly perceive as significant the possible therapeutic effects of engaging in creative activities for mentally ill persons.

**Table 1:** Participants’ profile and obtained frequency of study variables

Group number	Study groups	N	Variable frequencies									
			Existence of relationship		Relationship type		Creative person’s proneness to mental illness		Mentally ill more creative		Is creativity herapeutic?	
			yes	no	+ve	-ve	yes	no	yes	no	yes	no
1	Mental health professional	50	35	13	10	26	17	25	4	41	37	9
		(m = 27 f = 23)										
2	General public: Urban	200	135	49	22	109	48	139	17	171	148	42
		(m = 94, f = 106)										
3	General public- Rural	100	35	65	1	34	1	99	25	75	94	4
		(m = 51, f =49)										
	Total	350	205	12	33	169	66	263	46	287	279	55

**Table 2:** Chi Square analysis for study variables

Participant groups	Variables	Chi square			
		Value	df	Significance	Remarks
All groups	Relationship exists	18.3253	1	0.0000	Significant at 0.001 level. Significant perception of the existence of creativity—mental illness relationship
	Relationship type	91.5644	1	0.0000	Significant at 0.001 level. Significant perception of -ve relationship between
	Creative persons prone to mental illness	117.9605	1	0.0000	Significant at 0.001. Creative persons significantly perceived as not being prone to mental illness.
	Mentally ill persons more creative	174.4174	1	0.0000	Significant at 0.001. Mentally ill persons perceived significantly as not being more creative than others
	Creativity as therapy	150.2275	1	0.0000	Creativity significantly perceived as therapeutic for mental illness.
Mental health professional group	Relationship exists	10.0833	1	0.0015	Significant at 0.01 levels. Significant perception of the existence of creativity—mental illness relationship
	Relationship type	7.1111	1	0.0027	Significant at 0.01 level. Significant perception of -ve relationship between
	Creative persons prone to mental illness	1.5283	1	0.2170	Not significant.
	Mentally ill persons more creative	30.4222	1	0.0000	Significant at 0.001. Mentally ill persons perceived significantly as not being more creative than others
	Creativity as therapy	17.0435	1	0.0000	Creativity significantly perceived as therapeutic for mental illness.
General public: Urban	Relationship exists	40.1957	1	0.0000	Significant at 0.001 level. Significant perception of the existence of creativity—mental illness relationship
	Relationship type	57.7786	1	0.0000	Significant at 0.001 level. Significant perception of -ve relationship between
	Creative persons prone to mental illness	44.2834	1	0.0000	Significant at 0.001. Creative persons significantly perceived as not being prone to mental illness.
	Mentally ill persons more creative	26.1489	1	0.0000	Significant at 0.001. Mentally ill persons perceived significantly as not being more creative than others
	Creativity as therapy	59.1368	1	0.0000	Creativity significantly perceived as therapeutic for mental illness.
General public: Rural	Relationship exists	9.00	1	0.0027	Significant at 0.01 levels. Significant perception of non existence of creativity—mental illness relationship
	Relationship type	31.1143	1	0.0000	Significant at 0.001 level. Significant perception of -ve relationship between
	Creative persons prone to mental illness	96.0400	1	0.0000	Significant at 0.001. Creative persons significantly perceived as not being prone to mental illness.
	Mentally ill persons more creative	25.000	1	0.0000	Significant at 0.001. Mentally ill persons perceived significantly as not being more creative than others
	Creativity as therapy	82.6531	1	0.0000	Creativity significantly perceived as therapeutic for mental illness.

**Table 3:** Man Witney U test result of comparison of Rorschach ’O’ originality scores between schizophrenics and manic depressives

Groups	Rorschach ’O’ scores (Means)	U	W	Exact 2-tail P		Correction for ties	Remarks
		45.5	100.5	0.7394	z	2-tailed P	
						P	
Schizophrenics	1.6				-0.3580	0.7204	
Manic depressives	2.7						Not significant

Note: There is no significant difference between schizophrenics and manic-depressives on their scores for the Rorschach originality scores. However manic-depressives seem to score slightly more than others.

## Discussion

Mentally ill participants with a diagnosis of affective disorder did not show a significant increase in creativity compared with others who had a diagnosis of schizophrenia (although there was a slight positive difference). The research used a limited sample size and did not include a control group of people who did not have either schizophrenia or Affective Disorder. The reason for considering this finding interesting is that the relationship for which there exists at present the most positive evidence is that between creativity and Affective Disorder. The failure to find an association may be due to confounding factors listed above. The result may also be influenced by existence of symptoms of active illness in the patients surveyed.

The findings from the questionnaire survey showed that all the groups surveyed were unanimous in their belief that mental illness did not make people more creative than others, and any relationship that existed between mental illness and creativity was a negative one. All the groups also concluded that engaging in creative activities was therapeutically beneficial to persons with mental illness.

A finding of note is that although mental health professionals tended to believe that creative persons were more prone to mental disorders than others, members of the general public in both urban and rural populations felt that creative persons were not more prone to mental disorders than other people. This difference might be due to the greater level of interactional experience of mental health professionals with persons with psychological and psychiatric disorders. The general public on the other hand were mostly acquainted with the creative manifestation of creative individuals. Any psychopathological tendencies might be concealed from them.

Mental health practitioners and the urban sample surveyed significantly believed that there was a relationship between creativity and mental illness, whereas the rural sample believed that there was no significant relationship. This might be due to idolization by rural folk of the creative geniuses in their midst. Urban folk and mental health professionals might be able to have a more objective viewpoint stemming out of their higher level of education.

There was a general consensus that mental illness did not make anyone more creative, but that engaging in creative activity was beneficial for the recovery of the patient. The perception of the potential therapeutic effect of creative activity is of particular interest in the context of this study. The possible applications of this relationship abound in the design of therapeutic programmes for treatment of the mentally ill, but are not much recognised or used currently in the African environment. A useful item of cultural information in this connection is that traditional practitioners routinely use creative activities, such as music and drama, in the treatment and rehabilitation of mentally ill patients.

## Concluding Remarks

**Figure 1:**
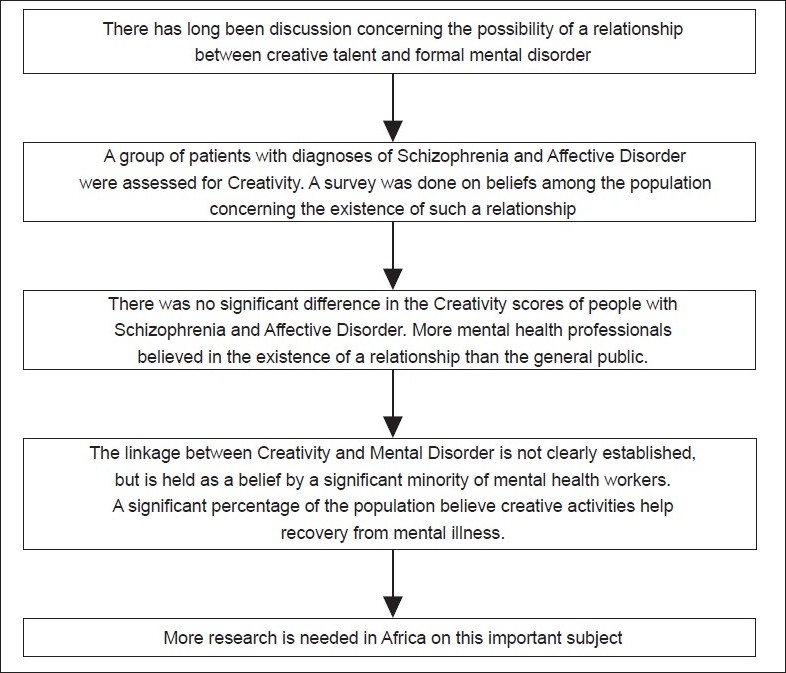
Flowchart of paper

The one area where there is uniformity of cultural opinion (the usefulness of creative activity in promoting patient’s recovery) is a useful area of future activity, by way of practice and research [Figure 1]. The information is not new, but the fact that it has widespread acceptance means that the people, including the patient and his relations, would have certain expectations of what environment is required for the promotion of recovery. Creating such an environment may not only directly influence the patient’s recovery positively, but also increase the level of cultural confidence such a person would have in the treatment system as a whole, given the fact that he has an alternative in traditional medicine.

### Take home message

The whole area of the relationship between creativity and mental disorder in the African context requires more active research. It would be interesting to do quantitative research to establish if the widespread view that creative activity can have a positive influence on recovery from psychotic illness is borne out by evidence.

## Questions Raised By The Paper

1. Are creative individuals truly recognised and valued in African (and other) societies?
2. Can creative talent be separated from such personality factors as personal force, dynamism, extroversion which make people stand out in the crowd?
3. Would the apparently general belief that creative activities can be therapeutic for the mentally ill be borne out by quantitative research?
4. Is the possession of creative ability a talent to be cherished, or evidence of a genetic flaw?
5. In a creative individual, does the existence of current illness potentiate creative expression, or diminish it? Are the processes of creativity similar to, or contradictory to, an illness process, such as a manic episode?

## About the Author

*Olufemi Olugbile is a Consultant Psychiatrist and hospital administrator whose main area of practice is Adult psychiatry. For the past decade, he has been based in Lagos Nigeria. His main areas of research interest include Social and Transcultural Psychiatry, focussing on such subjects as the influence of Culture on the understanding, manifestations and outcome of psychiatric illness, the nature and conceptual framework of tradition mental health practices, and the relationship between mental health and political behaviour. He has published a book on The Psychology of Work Among Nigerians, and has authored four other books on various topics, including fiction*

## About the Author

*Mathew Zachariah is a Clinical Psychologist with several years of experience, who has practiced in the Nigerian environment for many years. He shares a commonality of research interest with the first author, and has collaborated with him on a number of research projects, including a survey of attitudes concerning the compulsory mental health testing for politicians*

## References

[1] Adrian-Vallance E, Combley R, Stark M (2004). *Longman Active Study Dictionary*.

[2] Akinola M, Mendes W (2008). The dark side of creativity: biological vulnerability and negative emotions lead to greater artistic creativity, Personality and Social Psychology Bulletin.

[3] Andreasen N (1987). Creativity and Mental Illness; Prevalence rates in writers and their first degree relatives, *American Journal of Psychiatry*.

[4] Andreasen N (2008). The relationship between Creativity and Mood Disorders, *Dialogues in Clinical Neurosciences*.

[5] Andreasen N. C (2011). A Journey into Chaos: Creativity and the Unconscious. *Brain, Mind and Consciousness: An International, Interdisciplinary Perspective* (A.R. Singh and S.A. Singh eds.), MSM.

[6] Andreasen N, Glick I (1988). Bipolar Affective Disorder and Creativity: Implications and Clinic Management, *Comprehensive Psychiatry*.

[7] Brain W (1948). Some Reflections on Genius, *Eugenics Review*.

[8] Ellis H (1926). A Study of British Genius.

[9] Jamison K (1989). Mood Disorder And Patterns of Creativity in British writers and Artists, *Psychiatry*.

[10] Juda A (1949). The Relationship Between High Mental Capacity and Psychic Abnormalities, *American Journal of Psychiatry*.

[11] Lombroso C (1981). *The Man of Genius*.

[12] Ludwig A (1989). Reflections On Creativity And Madness, *American Journal of Psychotherapy*.

[13] MaCabe J, Lambe M.P, Sham P.C, Reichenberg A, Hultman C.M (2010). Excellent school performance at age 16 and risk of adult bipolar disorder: national cohort study, *British Journal of Psychiatry*.

[14] McNeil T (1971). Pre-birth and post-birth Influence on the relationship between creative ability and mental illness, *Journal of Personality*.

[15] Moses H (2010). Music and mental illness, *Annals of Neurology*.

[16] Murray G, Johnson L (2010). The clinical significance of creativity in bipolar disorder, *Clinical Psychology Review*.

[17] Richard S (1988). Creativity In Manic-Depressive, Cychothymes, their normal Relatives and Control subjects, *Journal of Abnormal Psychology*.

[18] Rust J, Golombok S, Abram S (1989). Creativity and Schizotypal Thinking, Journal of *Genetic Psychology*.

